# Behavioral decline and premature lethality upon pan-neuronal ferritin overexpression in *Drosophila* infected with a virulent form of *Wolbachia*

**DOI:** 10.3389/fphar.2014.00066

**Published:** 2014-04-04

**Authors:** Stylianos Kosmidis, Fanis Missirlis, Jose A. Botella, Stephan Schneuwly, Tracey A. Rouault, Efthimios M. C. Skoulakis

**Affiliations:** ^1^Neuroscience Division, Biomedical Sciences Research Centre “Alexander Fleming”Vari, Greece; ^2^Department of Neuroscience, Columbia UniversityNew York, NY, USA; ^3^Molecular Medicine Program, Eunice Kennedy Shriver National Institute of Child Health and Human Development, National Institutes of HealthBethesda, MD, USA; ^4^Departamento de Fisiología Biofísica y Neurociencias, Centro de Investigación y de Estudios Avanzados del Instituto Politécnico NacionalMéxico City, México; ^5^Institute of Zoology, University of RegensburgRegensburg, Germany

**Keywords:** metal, symbiosis, rickettsia, popcorn, *w*Mel, insect, immunity, aging

## Abstract

Iron is required for organismal growth. Therefore, limiting iron availability may be a key part of the host’s innate immune response to various pathogens, for example, in *Drosophila *infected with *Zygomycetes*. One way the host can transiently reduce iron bioavailability is by ferritin overexpression. To study the effects of neuronal-specific ferritin overexpression on survival and neurodegeneration we generated flies simultaneously over-expressing transgenes for both ferritin subunits in all neurons. We used two independent recombinant chromosomes bearing *UAS-Fer1HCH, UAS-Fer2LCH *transgenes and obtained qualitatively different levels of late-onset behavioral and lifespan declines. We subsequently discovered that one parental strain had been infected with a virulent form of the bacterial endosymbiont *Wolbachia*, causing widespread neuronal apoptosis and premature death. This phenotype was exacerbated by ferritin overexpression and was curable by antibiotic treatment. Neuronal ferritin overexpression in uninfected flies did not cause evident neurodegeneration but resulted in a late-onset behavioral decline, as previously reported for ferritin overexpression in glia. The results suggest that ferritin overexpression in the central nervous system of flies is tolerated well in young individuals with adverse manifestations appearing only late in life or under unrelated pathophysiological conditions.

## INTRODUCTION

Iron is essential for the growth of microorganisms and animals because it serves as a cofactor in many enzymes (Sheftel et al., 2012). Symbiotic relationships will therefore require the tuning of iron homeostasis between the organisms, whereas in parasitism, iron sequestration becomes an important factor in the antagonism between parasite and host (Cassat and Skaar, 2013). *Drosophila melanogaster* is used very widely to study basic biological questions and as a model of human disease (Rajan and Perrimon, 2013), but our knowledge of how iron homeostasis is maintained in *Drosophila* is still rather limited [reviewed in (Mandilaras et al., 2013; Tang and Zhou, 2013b)]. It is known that iron is normally stored in specialized intestinal cells expressing ferritin within their secretory system (Locke and Leung, 1984; Mehta et al., 2009). Iron-loaded ferritin can be excreted to the intestinal lumen and is also found in the hemolymph (Locke and Leung, 1984). Disrupted ferritin function by mutation or RNA interference (RNAi) results in embryonic or first instar larval lethality (Missirlis et al., 2007; Li, 2010; Tang and Zhou, 2013a). Overexpression of ferritin also leads to excess iron sequestration and functional iron deficiency, which does not impede development to adulthood (Missirlis et al., 2007; Gutierrez et al., 2013; Tang and Zhou, 2013a). Neuronal ferritin overexpression was found to be beneficial when flies were fed with aluminum (Wu et al., 2012), whereas glial ferritin overexpression resulted in ferritin-iron inclusions in a subset of glia of the optic lobes and mild behavioral defects with a late-onset of appearance (Kosmidis et al., 2011). However, the effects of neuronal ferritin overexpression in otherwise wild type individuals have not been studied to date and were the first objective of this study.

*Drosophila melanogaster* laboratory cultures commonly host a symbiotic relationship with the α-proteobacteria *Wolbachia *species, in which infected females show a reproductive advantage over non-infected, with no other overt fitness costs associated with the presence of the bacterium (Werren et al., 2008; Saridaki and Bourtzis, 2010). As an exception, a virulent *Wolbachia* strain causing degeneration and early death has been identified (Min and Benzer, 1997). Its name, *popcorn *(*w*MelPop) reflects the appearance of the bacteria visualized *in situ* with electron microscopy (EM) and is thought to result from its increased proliferation. The severity of *w*MelPop-mediated phenotypes has been shown to vary with ambient temperature and the genetic background of the host (Reynolds et al., 2003). A whole-genome sequence comparison between variants of *w*MelPop and *w*Mel revealed only minor genetic changes between the pathogenic and symbiotic strains, providing no direct clues to help explain differences in virulence (Woolfit et al., 2013). Intriguingly, a small genomic region encoding 24 bacterial genes (out of a total of 1,111 annotated in the species) was either triplicated or absent in the pathogenic strains (Woolfit et al., 2013). Finally, two recent imaging studies documented in anatomical detail the presence of *w*MelPop in the fly brain (Albertson et al., 2013; Strunov et al., 2013).

Previous results show that *Wolbachia* influences iron homeostasis in *D. melanogaster* and in the closely related species *D. simulans* (Brownlie et al., 2009; Kremer et al., 2009). When grown under iron-limiting conditions, *D. melanogaster *suffers reduced fecundity, but when infected with *w*Mel, the flies laid significantly more eggs in four out of six experimental trials (Brownlie et al., 2009). The beneficial effect of *w*Mel was only seen under iron stress conditions, but the likely relevance of these experiments were supported by the finding that two out of the three natural populations tested were iron-deficient as collected in the wild. In a different paradigm, where *Wolbachia *is an obligate parasite of the wasp *Asobara tabida*, a study identified that both ferritin chains were induced in non-infected wasps (Kremer et al., 2009). To investigate whether *Wolbachia* affected ferritin iron in other insect species, iron accumulation in infected and uninfected *D. simulans* was determined. No difference was seen under normal dietary conditions, but when iron was supplemented in the diet infected individuals accumulated significantly less iron. Therefore, it appears that in a variety of insect species the presence of *Wolbachia* enhances tolerance to iron stress. We describe here the effect of neuronal ferritin overexpression in infected and uninfected *D. melanogaster *adults as they age and provide the first example where imposing an iron stress exacerbates the virulence of a pathogenic form of *Wolbachia*.

## MATERIALS AND METHODS

### FLY STOCKS

The X-chromosome insertion of the pan-neuronal driver *elav-Gal4* was obtained from the Bloomington *Drosophila* Stock Center (#458). The construction of transgenic stocks *UAS-Fer1HCH* and *UAS-Fer2LCH* have been described in detail elsewhere (Missirlis et al., 2007). The *w*MelPop infection likely occurred from a balancer stock (not recorded) used to recover the *UAS-Fer1HCH*, *UAS-Fer2LCH *recombinant on the X-chromosome. *Wolbachia*-infected *D. simulans* flies were a kind gift from Kostas Bourtzis.

### MICROSCOPY

Adult brains were prepared, cut, and stained as described (Kretzschmar et al., 1997). Ultrathin Epon plastic sections were post stained with 2% uranyl acetate, followed by Reynolds’ lead citrate and stabilized for transmission electron microscopy (TEM) by carbon coating. Examination was done under an optical microscope or with a Zeiss EM10C/VR (Oberkochen, Germany) electron microscope at 40-80 kV.

### BEHAVIORAL ASSAYS

Female adult flies collected 0-2 days after eclosion were kept throughout their lifetime at 25°C in groups of 20 individuals per vial. For elicited-escape response tests flies were adapted for at least 20 min in an environment of 25°C and 70-80% humidity illuminated by red light. They were then placed in 14 mL polystyrene Falcon tubes individually and each fly was vortexed at the highest speed for 2-3 s and was tested twice in negative geotaxis and horizontal escape assays (Kosmidis et al., 2011). All flies were recorded for at least 1 min or until they reached their target of 10 cm. A minimum 20 flies per genotype were tested for each experimental point and the data analyzed parametrically for statistical significance using the JMP and Prism software. Lifespan determinations used a minimum of 120 flies per genotype.

### PCR-DETECTION OF *Wolbachia*

The presence of *Wolbachia* was determined by PCR using 16s rDNA *Wolbachia*-specific primers (Bourtzis et al., 1996). The primer sequences used were as follows: 5′-TTGTAGCCTGCTATG-GTATAACT-3′ and 5′-GAATAGGTATGATMTCATGT-3′.

## RESULTS

### PHENOTYPES OBSERVED BY FERRITIN OVEREXPRESSION IN ALL FLY NEURONS

**FIGURE 1:**
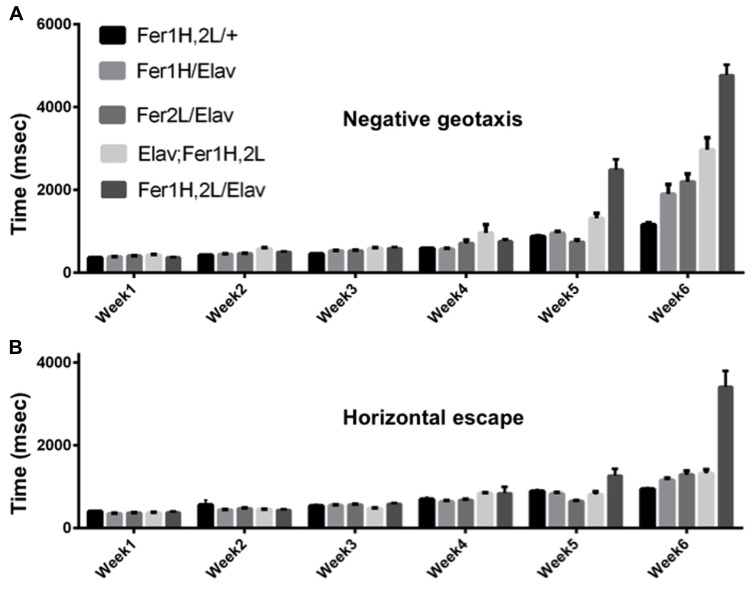
**Age dependent behavioral responses of flies overexpressing ferritin subunits pan-neuronally.** Elicited escape response of flies was assessed on a weekly basis. The first bar on each graph corresponds to control (uninfected from *Wolbachia*) flies carrying the ferritin subunit transgenes without the *elav-Gal4* driver. Bars 2 and 3 correspond to flies overexpressing *Fer1HCH* and *Fer2LCH*, respectively. Bars 4 and 5 correspond to flies simultaneously overexpressing both *Fer1HCH* and *Fer2LCH* from recombinant transgenes on the third and X chromosomes, respectively. **(A)** Flies expressing either the heavy or the light chain alone did not exhibit statistical differences from controls in their escape response until the fifth week and showed a moderate twofold increase in their response time on week 6 (*p* < 0.05). In contrast, flies overexpressing both ferritin subunits showed a significant impairment at 5 and 6 weeks of age (*p* < 0.001). **(B)** Only flies overexpressing both ferritin subunits from the X-chromosome were significantly compromised when responding in the horizontal direction in weeks 5 and 6 (*p* < 0.001).

We used the pan-neuronal driver *elav-Gal4* to overexpress both ferritin subunits simultaneously from recombinant chromosomes bearing transgene insertions of *UAS-Fer1HCH *and *UAS-Fer2LCH.* As the X-chromosome was modified in one stock to carry the two *UAS* transgenes and in the other stock to carry the *Gal4* insertion in the *elav *locus, we used only female flies in our experiments. Following eclosure, adult females were kept with their male siblings for 2 days to ensure successful mating and thereafter, female cohorts were kept under non-crowded conditions with biweekly transfers to fresh food. Flies bearing the transgenes alone were used as controls, but we note that *UAS-Fer1HCH, UAS-Fer2LCH*/+; +/+; +/+ females behaved differently depending on whether their mothers were infected with *Wolbachia *or not and this observation (reported further below) was unknown to us when experiments were originally planned and performed. In **Figure 1** our controls were derived from uninfected *white *(*w*) mutant mothers. We also tested the effect of neuronal overexpression of single subunits, as they have been previously shown to rescue degeneration associated with soluble β-amyloid overexpression (Rival et al., 2009). Once weekly during the lifespan of the flies, we monitored the time required for individual flies to climb 10 cm following a brief stimulus that elicited a negative geotactic response (**Figure 1**). The control flies used in this experiment showed a modest two-fold increase (*p* < 0.05) in the time required to perform this test during the sixth week of testing. Flies overexpressing either ferritin subunit alone behaved similar to controls through the fifth week, but presented significant performance impairment (a fourfold increase in time, *p* < 0.001) when the aging-related decline initiated at week six. The flies overexpressing both ferritin subunits from the transgenes on the third chromosome showed a modest compromise at weeks 5 and 6 (threefold and sixfold increases; *p* < 0.0001). The flies overexpressing both ferritin subunits from the transgenes on the X chromosome showed a robust mobility impediment one week earlier than controls (fourfold increase in time by week five, *p* < 0.001), with a dramatic tenfold increase in the time required to climb 10 cm by week six (*p* < 0.0001; **Figure 1A**). Similar findings were found when testing horizontal escape responses, which presumably require less physical effort from individual flies (**Figure 1B**). During their sixth week post-eclosion, *elav-Gal4/UAS-Fer1HCH, UAS-Fer2LCH; *+/+; +/+ flies could hardly walk.

**FIGURE 2:**
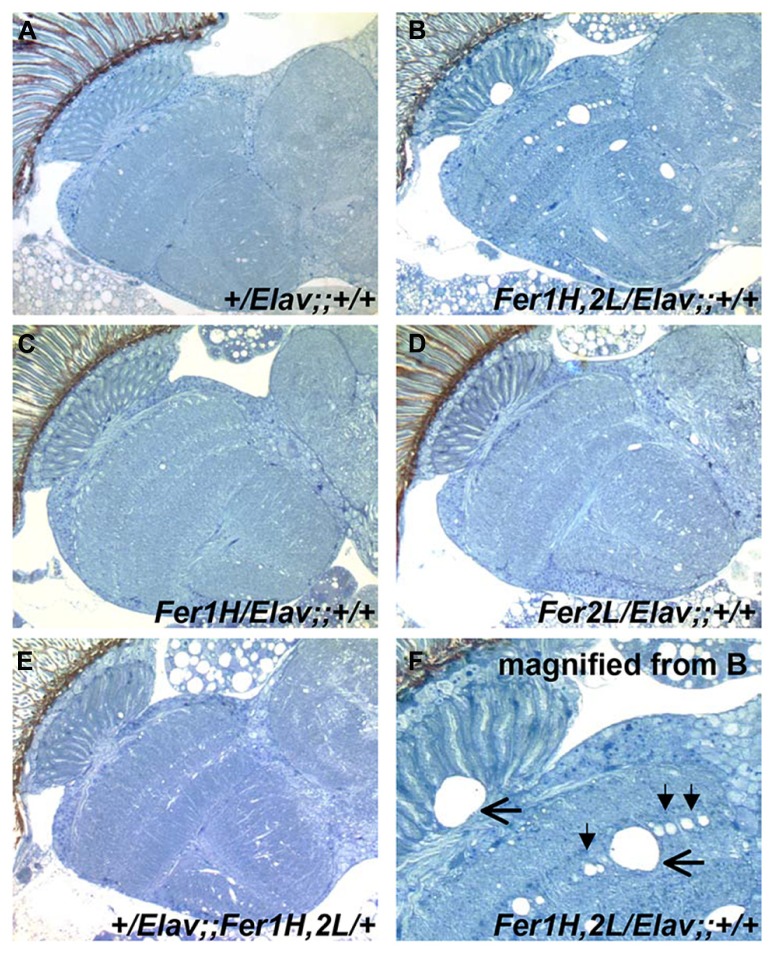
**Plastic embedded sections from 40-day old fly heads stained with toluidine blue.** **(A)** Control flies show no signs of degeneration at this age (grown at 25°C). **(B) **Flies overexpressing ferritin derived from *UAS-Fer1HCH, UAS-Fer2LCH*; +; + mothers crossed to *Elav-Gal4; *+; + fathers show extensive neurodegeneration. **(C,D)** Flies overexpressing single ferritin subunits show no signs of neurodegeneration. **(E)** Flies overexpressing ferritin from the recombinant insertions on the third Chromosome also show no apparent neurodegeneration. **(F)** Close-up of image shown in **(B)**. Filled arrowheads denote the absence of single neuronal cell bodies lined up in the optic medulla. Horizontal arrows indicate areas of extensive neuronal damage.

**FIGURE 3:**
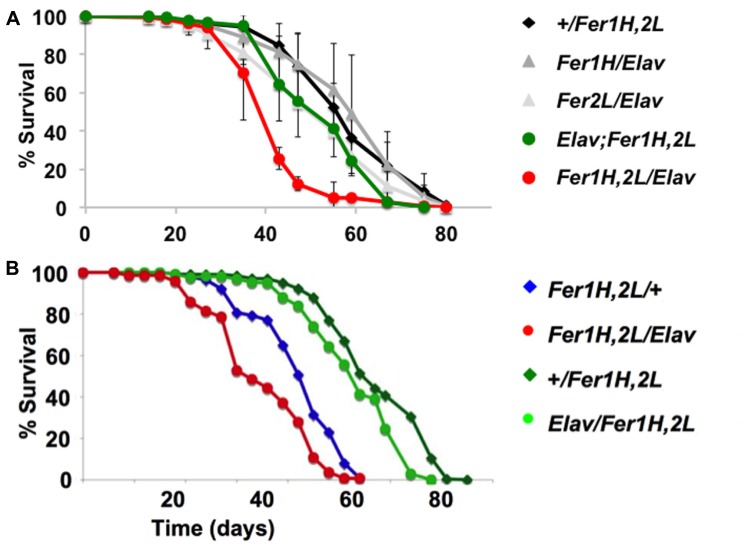
**Lifespan determinations of cohorts of female adult *D. melanogaster.*** Day 0 marks eclosion, flies were kept with the opposite sex for the first five days, then they were separated on groups of 20 and transferred to fresh food regularly. **(A)** The same genotypes assayed in **Figure 1** for behavior were used here. The experiments were performed three times independently (each time with a minimum cohort of 120 flies per genotype) and standard deviations are shown between the three experiments. Note the early onset of mortality in the flies overexpressing ferritin from the X-chromosomal recombinant. **(B)** A similar single experiment demonstrating that a maternal component determines the early onset mortality. Note that the same genotypes have markedly different lifespans, whose length is predicted by the mothers used for the crosses (see text for details). Overexpression of ferritin was never seen to have a beneficial effect on lifespan.

We then prepared plastic-embedded sections of adult fly heads to monitor the neuroanatomical integrity of the brains of 40-day old flies, at the transition between weeks 5 and 6. No apparent degeneration was evident in *elav-Gal4*/+; +/+; +/+ controls (**Figure 2A**), flies overexpressing the single ferritin subunits (**Figures 2C,D**) and those overexpressing ferritin from the third chromosomal recombinant (**Figure 2E**). In stark contrast, animals overexpressing ferritin from the X-chromosome showed severe neuronal degeneration, evident throughout the brain, including the optic lobes (**Figures 2B,F**). Furthermore, lifespan determinations, clearly distinguished between ferritin overexpressing flies derived from the third and X-chromosomal recombinants, with the latter presenting precipitous mortality during the sixth week of aging, 2 weeks earlier than controls (**Figure 3A**). Therefore, in addition to the significant difference in the onset of the mobility impediment between the two genotypes of flies overexpressing both ferritin subunits, brain pathology, and reduced lifespan were also associated only with the ferritin overexpression induced from the X-chromosomal transgenes.

### MATERNAL EFFECT ASSOCIATED WITH REDUCED LIFESPAN OF FERRITIN OVEREXPRESSING FLIES

To investigate the potential cause of the differential shortened lifespan, neurodegeneration and early onset behavioral decline of the X-chromosome driven ferritin overexpression, we tested whether there was a maternal effect associated with these phenotypes. We crossed homozygous female *UAS-Fer1HCH, UAS-Fer2LCH* to male *elav-Gal4* or to* w *control flies and *vice-versa*. *UAS-Fer1HCH, UAS-Fer2LCH/elav-Gal4* flies were therefore derived from *UAS-Fer1HCH, UAS-Fer2LCH* or *elav-Gal4* mothers, whereas *UAS-Fer1HCH, UAS-Fer2LCH/*+ control flies were derived from *UAS-Fer1HCH, UAS-Fer2LCH,* or *w* mothers. The results clearly show that lifespan shortening was observed only in progeny of *UAS-Fer1HCH, UAS-Fer2LCH *mothers (**Figure 3B**). In agreement with this notion the decrease was also seen in control *UAS-Fer1HCH, UAS-Fer2LCH/*+ animals that do not overexpress ferritin since they do not carry the *elav-Gal4 *driver, but their transgene carrying chromosome is maternal in origin. Therefore, the behavioral, neurodegenerative, and reduced life span phenotypes are not associated with over-expression of the ferritin subunits, but rather with the maternal origin of the transgene-bearing chromosome.

### *Wolbachia* CLUSTERS DETECTED BY TEM AND PCR IN THE SEVERELY AFFECTED FERRITIN OVEREXPRESSING FLIES

**FIGURE 4:**
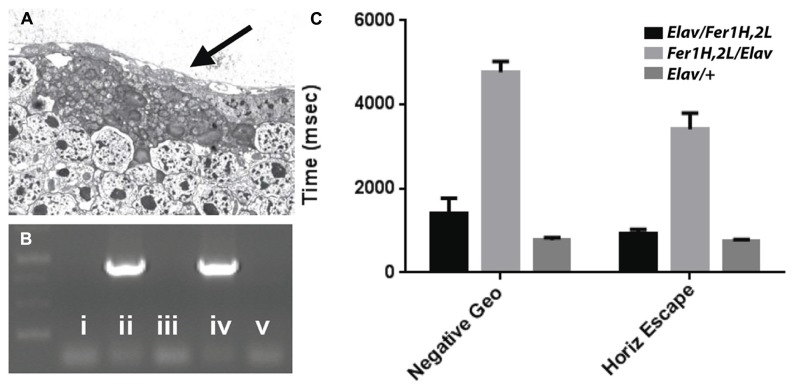
**(A)** Bacteria accumulating amongst neurons and bearing the characteristic popcorn appearance of the *w*MelPop strain. **(B)** PCR detection of the *Wolbachia* ribosomal 16S gene in the following genotypes (i) no DNA, (ii) *UAS-Fer1HCH, UAS-Fer2LCH *recombinant on X, (iii) *elav-Gal4* on X, (iv) *UAS-Fer1HCH, UAS-Fer2LCH/elav-Gal4* (mothers from ii), (v) *Elav-Gal4/UAS-Fer1HCH, UAS-Fer2LCH* (mothers from iii). **(C)** Behavioral responses of (v) – black bars – *Elav-Gal4/*UAS-Fer1HCH, UAS-Fer2LCH, (iv) – light gray bars – *UAS-Fer1HCH, UAS-Fer2LCH/elav-Gal4* and *Elav-Gal4*/+ control flies (dark gray bars) at 6 weeks of age.

**FIGURE 5:**
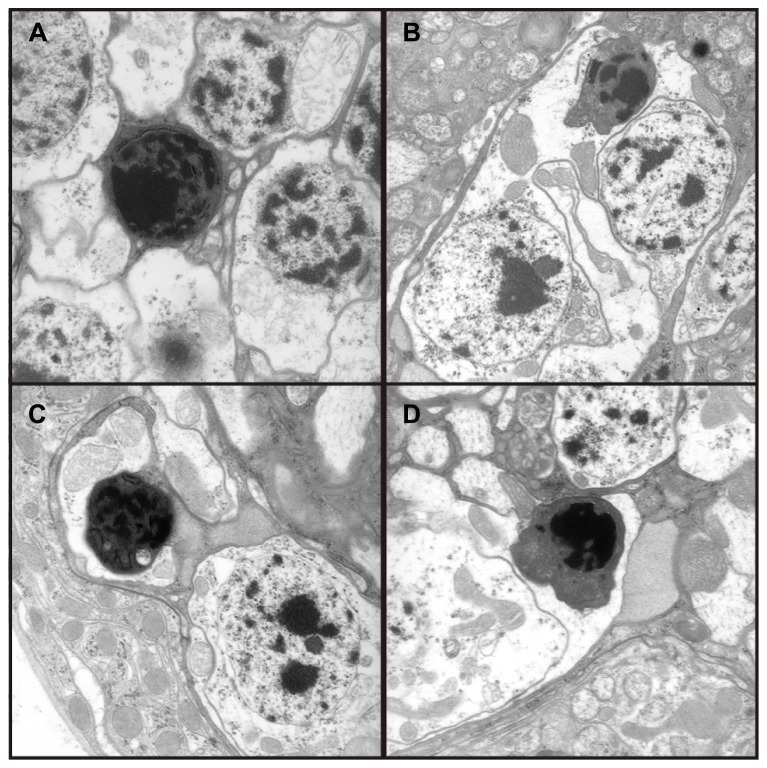
**(A–D)** Four independent electron micrographs from *Wolbachia* infected flies showing characteristic apoptotic nuclei, suggesting neuronal death results from apoptosis.

**FIGURE 6:**
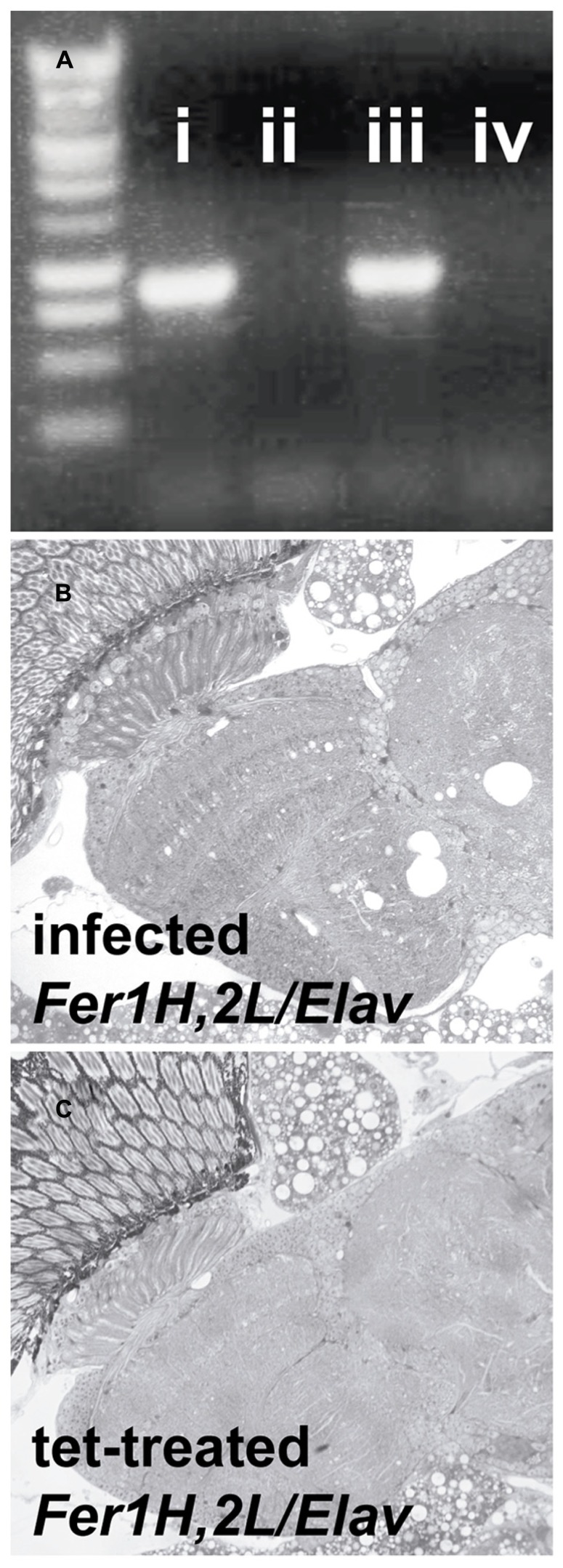
**Treatment with the antibiotic tetracycline cured the flies from the *Wolbachia* infection.** **(A)** PCR detection of the *Wolbachia* ribosomal 16S gene in (i) infected *D. simulans* (ii) tetracycline-treated *D. simulans *(iii) infected *UAS-Fer1HCH, UAS-Fer2LCH *on X (*D. melanogaster*) (iv) tetracycline-treated *UAS-Fer1HCH, UAS-Fer2LCH* on X **(B)** Plastic embedded sections of heads from infected (mothers from (iii) and **(C)** tetracycline-treated (mothers from iv) *Elav/UAS-Fer1HCH, UAS-Fer2LCH* adults at 40 days of age.

Transmission electron microscopy images from affected *UAS-Fer1HCH, UAS-Fer2LCH/elav-Gal4* flies revealed the presence of bacterial infections, with the characteristic morphology attributed to *w*MelPop (**Figure 4A**; Min and Benzer, 1997; Strunov et al., 2013). To verify the presence of *Wolbachia* independently, we performed genomic PCR using bacteria-specific primers on the *UAS-Fer1HCH, UAS-Fer2LCH* and *elav-Gal4* parental lines and on* UAS-Fer1HCH, UAS-Fer2LCH/elav-Gal4* progeny derived from presumably infected and uninfected mothers (**Figure 4B**). We verified the presence of *Wolbachia* in mothers and progeny derived from the stock bearing the *UAS-Fer1HCH, UAS-Fer2LCH* recombinant on the X-chromosome. We also assessed negative geotaxis on *UAS-Fer1HCH, UAS-Fer2LCH/elav-Gal4 *flies and in agreement with the life span experiment, we only observed a large time delay in infected individuals at 5 weeks of age (**Figure 4C**). Although the severe neurodegeneration that follows *w*MelPop infection has been previously documented, it has been assumed that the bacterial infection leads to neuronal necrosis (Min and Benzer, 1997). Our TEM analysis demonstrated numerous pyknotic nuclei within CNS neurons (**Figure 5**), consistent with the characteristic nuclear changes that accompany programmed cell death. Treatment of the *UAS-Fer1HCH, UAS-Fer2LCH* stock with the antibiotic tetracycline for four consecutive generations eliminated the infection (**Figure 6A**). *UAS-Fer1HCH, UAS-Fer2LCH/elav-Gal4 *flies derived from the tetracycline-treated *UAS-Fer1HCH, UAS-Fer2LCH* mothers retained their brain integrity at 40 days of age (**Figures 6B,C**) and, by qualitative observations, showed no signs of premature mobility decline and mortality.

## DISCUSSION

### IRON AND *Wolbachia* SYMBIOSIS

The maternally inherited bacterium *Wolbachia* infects arthropods and nematodes (Serbus et al., 2008). In filarial nematodes, *Wolbachia *is an obligate symbiont required for the successful reproduction of the worm (Bandi et al., 1999). The hypothesis that *Wolbachia* contributes metabolic provisioning to its hosts originated from whole genome sequencing projects showing that the entire heme biosynthesis pathway was present in *Wolbachia* but absent in *Brugia malayi* (Foster et al., 2005). This hypothesis was tested in *D. melanogaster* supporting the notion that the presence of *Wolbachia* assisted the flies under conditions of iron stress (Brownlie et al., 2009). Further support to the idea that iron is important in symbiotic relationships of *Wolbachia* and its hosts came from the finding that ferritin expression was induced in the absence of *Wolbachia* from *A. tabida *(Kremer et al., 2009). However, we still have no molecular understanding of the iron-dependent interactions between *Wolbachia* and its hosts. The same is true for interactions of *Wolbachia* with other metals (Wang et al., 2012) or with the host’s antioxidant defense systems (Brennan et al., 2008).

Our study provided evidence that *w*MelPop, or a *w*MelPop-like strain, causes neuronal death, likely by inducing the apoptotic pathway in infected brains, with apoptosis seen also in the optic lobes, which generally were found to harbor significantly less bacteria (Albertson et al., 2013; Strunov et al., 2013). Ferritin overexpression, which likely results in depletion of readily bioavailable iron (Gutierrez et al., 2013), conferred no apparent protection to infected flies, but instead further decreased their shortened lifespan due to *Wolbachia* infection (**Figure 3B**). Our experiments did not discriminate between the possibility that ferritin overexpression benefited the pathogen, encouraging its propagation, or, alternatively, that ferritin overexpression on top of pathogen infection further deprived bioavailable brain iron. Our results point to a different outcome between *w*MelPop and *w*Mel infections when responding to iron deficiency, with the former killing their host more effectively when the latter appear to provide an advantage to their host (Brownlie et al., 2009), although our observations call for further, differently controlled, experiments to settle this question. It will be of interest to investigate whether iron-related genes are included in the genomic region that appears to distinguish the pathogenic *w*MelPop strains from the more common endosymbiotic *w*Mel variants (Woolfit et al., 2013).

### IRON LIMITATION AND INSECT IMMUNITY

The clearest evidence that iron availability can determine the outcome of an infection in *Drosophila* was provided by a study of infection with the human fungal pathogen *Zygomycetes* (Chamilos et al., 2008). Provision of iron to the fungus resulted in more aggressive infections whereas treatment of flies with an iron chelator suppressed the infection. This outcome is consistent with the broadly recognized role of iron in infection: its availability is good for the pathogen and bad for the host (Drakesmith and Prentice, 2012). Although very limited studies have been conducted investigating the role of iron during infections in *Drosophila* to date, the emerging picture appears to be complex and specific to each microbe studied. Hence, three qualitatively different interactions have been noted: (i) that iron limitation stops an infection (Chamilos et al., 2008), (ii) that iron limitation exacerbates the pathogenesis of an infection (this study), or (iii) that symbiotic infection becomes beneficial to the host under iron limitation (Brownlie et al., 2009). Clearly the role of iron in *Drosophila* immunity is an exciting open field for study.

### FERRITIN OVEREXPRESSION IN THE CENTRAL NERVOUS SYSTEM OF *Drosophila*

We previously described the effect of ferritin overexpression in all *Drosophila *glia (Kosmidis et al., 2011) and in terms of behavior and lifespan, the major conclusions are very similar to these reported here for neurons: there seems to be no decrease in lifespan of flies overexpressing ferritin and there is only a small but readily quantifiable late-onset behavioral defect in negative geotactic responses. Although there is a detectable negative effect from ferritin overexpression at old age in wild type conditions, it has been clearly shown that ferritin overexpression can be protective under conditions of metal stress (Missirlis et al., 2007; Wu et al., 2012). Ferritin overexpression is thought to generate an iron limitation, but ferritin may be also able to sequester other metals with a significantly lower capacity (Gutierrez et al., 2013). One particularity of overexpressing ferritin in glia is that iron-loaded ferritin accumulated in a subset of glia in the optic lobes forming inclusions (Kosmidis et al., 2011). No such accumulation was detected with ferritin overexpression in neurons. In addition, RNAi experiments against each ferritin subunit resulted in behavioral alterations when neurons were targeted and lethality when ferritin was suppressed in glia (Mandilaras and Missirlis, 2012; Tang and Zhou, 2013a). Our results suggest that ferritin overexpression, and iron chelators more generally (Soriano et al., 2013), should be considered as a therapeutic means of intervention, but a more general recommendation for iron chelation in otherwise healthy adults is not supported from the model organism *D. melanogaster*.

## AUTHOR CONTRIBUTIONS

All authors designed the original set of experiments aiming to address the consequences of ferritin overexpression in neurons of wild type flies and participated in the data analysis. Fanis Missirlis generated the transgenic flies and measured the life spans. Stylianos Kosmidis performed the behavioral tests, the molecular biology to detect *Wolbachia* and the antibiotic treatments. Jose A. Botella and Stephan Schneuwly performed the electron microscopy analysis. Tracey A. Rouault and Efthimios M. C. Skoulakis had continuous input during each stage of the project, including during the writing of the manuscript, with Fanis Missirlis in a coordinating role.

## Conflict of Interest Statement

The authors declare that the research was conducted in the absence of any commercial or financial relationships that could be construed as a potential conflict of interest.
